# Biomechanical comparison of anterior cervical corpectomy and fusion, anterior controllable antedisplacement and fusion, and anterior cervical X-shape-corpectomy and fusion in the surgical treatment of ossification of the posterior longitudinal ligament: a finite element analysis

**DOI:** 10.3389/fbioe.2025.1594016

**Published:** 2025-11-11

**Authors:** Xiong-Han Lian, Huo-Huo Xue, Wen-Jia Sun, Yu-Fan Chen, Zhi-Feng Zeng, Liang Chen, Jing-Lai Xue

**Affiliations:** 1 Fuzhou Second General Hospital, Fuzhou, China; 2 Fujian University of Traditional Chinese Medicine, Fuzhou, China; 3 The First Affiliated Hospital of Fujian Medical University, Fuzhou, China

**Keywords:** anterior cervical corpectomy and fusion, anterior controllable antedisplacement and fusion, anterior cervical X-shape-corpectomy and fusion, ossification of the posterior longitudinal ligament, finite element

## Abstract

**Background:**

Anterior Cervical Corpectomy and Fusion (ACCF), Anterior Controllable Antedisplacement and Fusion (ACAF), and Anterior Cervical X-Shape-Corpectomy and Fusion (ACXF) have been shown to achieve similar decompression outcomes in the treatment of ossification of the posterior longitudinal ligament. However, the potential biomechanical differences remain unclear.

**Methods:**

Finite element models of the cervical spine (C3-C7) were constructed to simulate ACCF, ACAF, and ACXF. Compare the ranges of motion (ROMs), von Mises stresses in the fixation systems and cortical endplates, and adjacent intervertebral disc pressures (IDPs) under loading conditions.

**Results:**

Postoperatively, ROMs in the fusion area were significantly restricted, with ACAF exhibiting the most severe, followed by ACCF, while ACXF showed the lightest. Peak stresses in the internal fixation systems were highest in ACCF, particularly within the fusion devices. The cages in ACAF experienced lower stress than those in ACXF, whereas the screws showed the opposite trend. ACCF had the highest cortical endplate stresses, while ACXF had the lowest adjacent IDPs.

**Conclusion:**

ACAF and ACXF demonstrate superior biomechanical properties in terms of stability, reduced internal fixation system risk, resistance to subsidence, and lower incidence of adjacent segment disease. As a result, they may serve as viable alternatives to ACCF in certain cases.

## Introduction

Ossification of the posterior longitudinal ligament (OPLL) was first reported by Key in 1838 and further described in detail by Tsukimoto in 1960 (Hao et al., 2017). The exact pathogenesis of OPLL remains unclear, but it is generally believed to be influenced by genetic and environmental factors. Epidemiological studies indicate that the prevalence of OPLL in the Japanese population aged >20 years ranges from 1.9% to 4.3%, while in Europe and North America, it is between 0.1% and 1.7% (Matsunaga and Sakou, 2012). Approximately 70% of cases occur in the cervical region, with the thoracic and lumbar regions accounting for 15% each (Matsunaga and Sakou, 2012; Saetia et al., 2011; Kawaguchi et al., 2013). C5 is the most commonly affected vertebra (Matsunaga and Sakou, 2012; Saetia et al., 2011; Fujimori et al., 2016; Kawaguchi et al., 2016). Imaging studies have shown that the cervical OPLL (COPLL) detection rate in the Japanese population can be as high as 6.3% (Fujimori et al., 2016) compared to only 1.6% in non-Asian populations (Fujimori et al., 2015).

COPLL can lead to secondary spinal canal stenosis, resulting in compression of the spinal cord. It severely impairs the quality of life and may even result in the loss of the ability to perform activities of daily living. Conservative treatments are frequently ineffective, and follow-up studies have shown that ossification can progress both transversely and longitudinally (Wang et al., 2019). Therefore, surgical intervention is crucial for relieving spinal cord compression, restoring the physiological curvature of the cervical spine, and facilitating neurological recovery (Sun et al., 2020).

Surgical approaches for COPLL are generally classified into anterior and posterior approaches. While anterior surgeries are more technically challenging and carry higher risks, they offer superior decompression and improved postoperative recovery (Feng et al., 2016; Wang et al., 2017). Newer techniques, such as Anterior Controllable Antedisplacement and Fusion (ACAF) and Anterior Cervical X-Shape-Corpectomy and Fusion (ACXF) (Sun et al., 2017; Yang et al., 2018; Liu et al., 2021; Wang H. et al., 2023), are similar to the classic Anterior Cervical Corpectomy and Fusion (ACCF) procedure in that they can remove the ossified tissue in the posterior aspect of the vertebrae to achieve complete decompression. However, the biomechanical differences among these approaches remain unclear.

Finite element (FE) analysis is a numerical method that subdivides a structure into finite elements and applies physical laws for simulation and calculation. It has been widely used in biomechanical studies of complex medical scenarios. In this study, the FE model of COPLL was developed to simulate the surgical procedures of ACCF, ACAF, and ACXF. By analyzing the ranges of motion (ROMs), von Mises stresses in internal fixation systems and cortical endplates, and adjacent intervertebral disc pressures (IDPs). This study aims to evaluate the biomechanical effects and provide theoretical insights for clinical practice.

## Methods

### Establish EF models

The FE model was developed using high-resolution continuous thin-slice CT data from a healthy 52-year-old male volunteer (height: 168 cm; weight: 70 kg; supine position) with no history of spine-related diseases, such as fractures, deformities, or tumors. This study was approved by the Ethics Committee of Fuzhou Second General Hospital, and informed consent was obtained from the volunteer.

First, DICOM-format CT data were imported into Mimics Medical (version 21.0; Materialise Mimics, Leuven, Belgium), where thresholding was applied to extract the bony structures of C3-C7. Next, Geomagic Wrap (version 2021; Geomagic, Research Triangle Park, North Carolina, United States) was used for mesh reconstruction, surface smoothing, and patch division. SOLIDWORKS (version 2020; Dassault Systems SOLIDWORKS Corp, Waltham, MA, United States) was then employed to model and assemble the cortical bone, trabecular bone, annulus fibrosus, nucleus pulposus, endplate, facet cartilage, and fixation system (the screw: 14 mm long, 3 mm diameter; the titanium mesh: 26 mm high, 10 mm inner diameter, 12 mm outer diameter, cylinder; the titanium plate: 1 × 14 × 38 mm; the cage: 5 × 14 × 16 mm). The intervertebral disc consists of the annulus fibrosus and nucleus pulposus, with a volume ratio of 6:4. Annulus fibers surrounded the ground substance with an inclination to the transverse plane between 15° and 30°, accounting for approximately 19% of the entire annulus fibrosus volume (Zhang J. et al., 2023). The cortical bone, endplate, and facet cartilage were modeled with a thickness of 0.5 mm (Zhang J. et al., 2023; Lin et al., 2024). Finally, springs were used in ANSYS Workbench (version 2022 R2; ANSYS, Pennsylvania, United States) to simulate the ligament complex. All material properties were assumed to be homogeneous and isotropic, with relevant values for Young’s modulus and Poisson’s ratio provided in Table 1 (Lin et al., 2024; Shen et al., 2022).

**TABLE 1:** Spinal structure and instrumentation material properties.

Spinal structure and instrumentation	Young’s modulus (MPa)	Poisson’s ratio
Vertebral body		
Cortical bone	12,000	0.30
Cancellous bone	450	0.30
Endplate	500	0.40
Intervertebral disc		
Fibers	110	0.30
Ground substance	4.2	0.49
Nucleus pulposus	1.0	0.49
Facet joint cartilage	10.4	0.40
Ligament		
Anterior longitudinal ligament	10	0.30
Posterior longitudinal ligament	10	0.30
Capsular ligament	10	0.30
Interspinous ligament	1.5	0.30
Supraspinal ligament	1.5	0.30
Ligamentum flavum	1.5	0.30
Implants		
Titanium (plate, screw, and mesh)	110,000	0.30
PEEK (cage)	3,600	0.30

### Surgical procedures

#### ACCF

**FIGURE 1:**
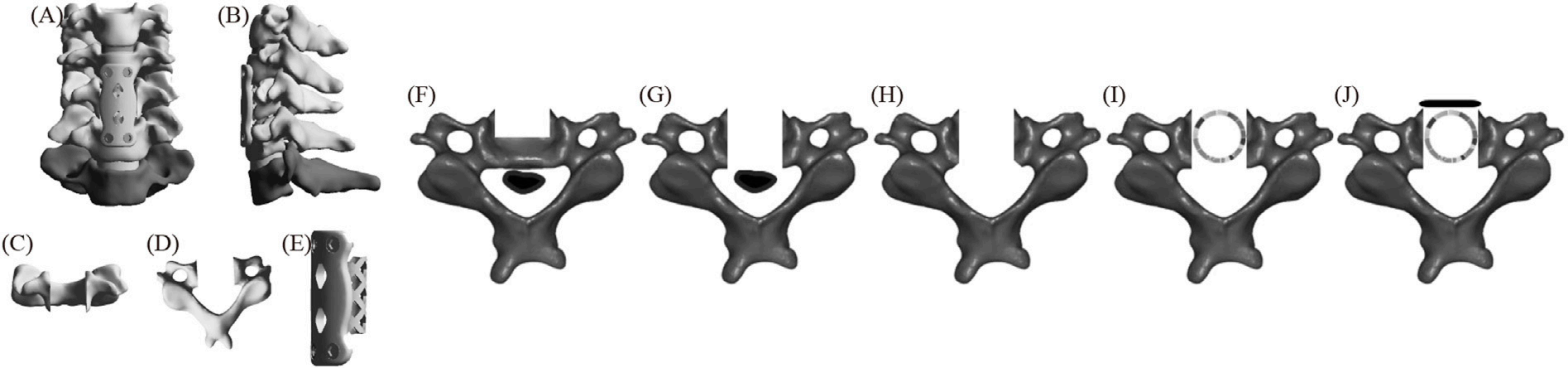
The FE model of ACCF **(A)** positive position; **(B)** lateral position; **(C)** C5 positive position; **(D)** C5 upper position; **(E)** fixation system; **(F–J)** C5 surgical procedure.

Figure 1 displays the FE model of ACCF. First, the intervertebral discs and cartilage endplates at C4/5 and C5/6 were resected. Subtotal resection of C5 was then performed, followed by decompression of the ossified tissue. Next, a titanium mesh was installed between C4 and C6, close to the residual vertebrae of C5. Finally, a plate spanning C4-C6 was positioned along the anterior edge and fixed with two screws at the proximal and distal ends, respectively, to ensure stability.

#### ACAF

**FIGURE 2:**
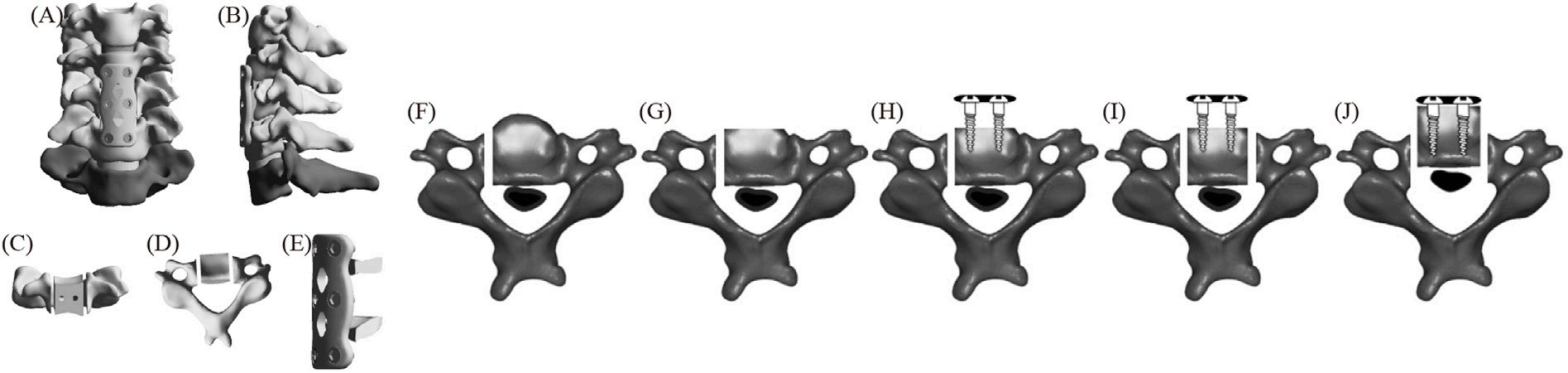
The FE model of ACAF **(A)** positive position; **(B)** lateral position; **(C)** C5 positive position; **(D)** C5 upper position; **(E)** fixation system; **(F–J)** C5 surgical procedure.

Figure 2 displays the FE model of ACAF (Sun et al., 2017). First, the intervertebral discs and cartilage endplates at C4/5 and C5/6 were resected. A slot was then created on the C5 side, and the anterior bone was partially resected. Next, cages were placed at C4/5 and C5/6, respectively. A plate spanning C4-C6 was positioned along the anterior edge and fixed with two screws at each vertebral body. All screws, except at C5, were tightened. Finally, a second slot was created on the opposite side of C5, and the vertebrae-OPLL complex was displaced forward by tightening the screws.

#### ACXF

**FIGURE 3:**
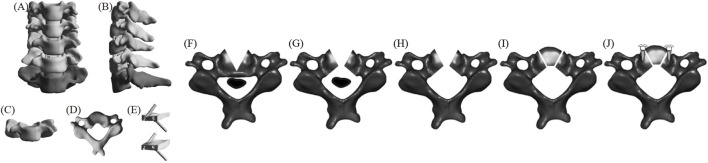
The FE model of ACXF **(A)** positive position; **(B)** lateral position; **(C)** C5 positive position; **(D)** C5 upper position; **(E)** fixation system; **(F–J)** C5 surgical procedure.

Figure 3 displays the FE model of ACXF (Wang H. et al., 2023). First, the intervertebral discs and cartilage endplates at C4/5 and C5/6 were resected. A V-shaped osteotomy was then performed from both sides of C5 towards the center, with the confluence at the posterior part of the vertebral body and the angle between the cross-section and the sagittal plane is approximately 25°. A second inverted V-shaped osteotomy was performed towards the posterior edge of C5. Next, the ossified tissue was removed, and the bone block was transplanted back. Finally, zero-profile cages were installed at C4/5 and C5/6, with two screws inserted obliquely at each level.

### Convergence analysis

Convergence analysis is a method of evaluating the accuracy of the FE model by increasing the mesh density to obtain stable results. The percentage changes in the peak von Mises stress were assessed at four different grid sizes (2, 1.5, 1, and 0.5 mm). The final grid size was 1 mm, which satisfied the peak von Mises stress variation range of <5% while reducing computational cost (Zhang et al., 2022). The relevant data are listed in Table 2.

**TABLE 2:** Convergence analysis results.

Element size	Nodes	Units	Percentage change
2.0 mm	241,351	125,567	>5%
1.5 mm	330,722	175,067	>5%
1.0 mm	589,736	326,699	<5%
0.5 mm	2,601,843	1,617,148	<5%

### Contact, boundary, and load conditions

Contact relationships exist between adjacent structures. The articular surfaces of the facet joints are considered frictionless (Zhang J. et al., 2023; Lin et al., 2024; Shen et al., 2022). Tie constraints are applied between the implant system and the cervical spine structures to simulate rigid fusion and ensure adequate osseointegration (Zhang J. et al., 2023; Shen et al., 2022). The model is fixed by constraining the movement of the lower endplate of C7 in all directions, while C3 is unrestricted. A vertical load of 73.6 N is applied to the upper endplate of C3 to simulate the weight of the head, along with a torque of 1.0 Nm to facilitate flexion, extension, lateral bending, and axial rotation (Ahn et al., 2023; Wang H. et al., 2023).

## Result

### Validity verification

**FIGURE 4:**
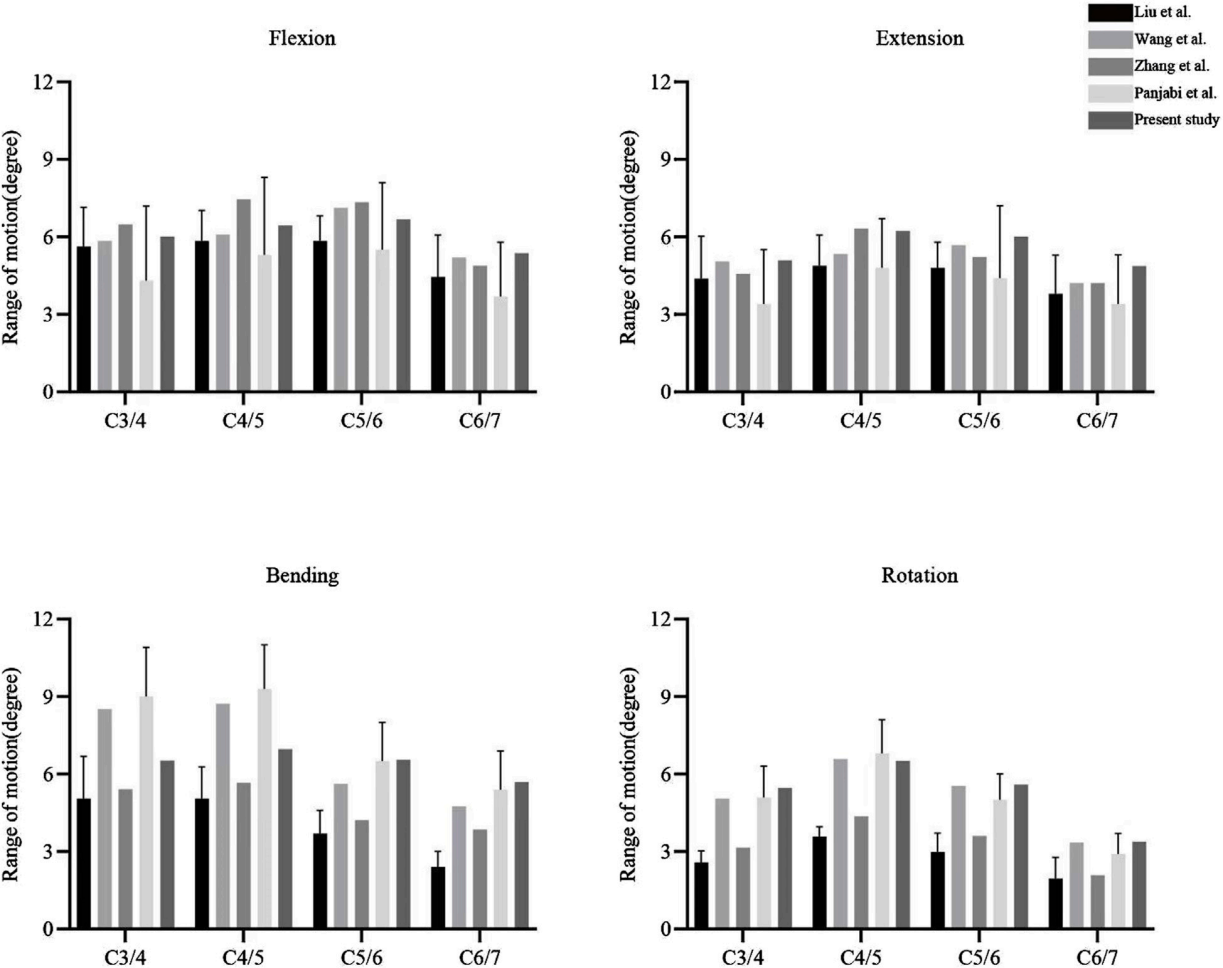
Comparison of ROMs at C3-7 between published researches and present study.

The ROMs of the FE model were compared with published results (Zhang et al., 2022; Wang Y. et al., 2023; Panjabi et al., 2001; Liu et al., 2016). The ROMs at C3/4, C4/5, C5/6, and C6/7 were measured as 6.01°, 6.44°, 6.68°, and 5.37° in flexion; 5.09°, 6.23°, 6.01°, and 4.87° in extension; 6.52°, 6.96°, 6.55°, and 5.69° in lateral bending; and 5.46°, 6.51°, 5.59°, and 3.38° in axial rotation, respectively (Figure 4). These results fell within the standard deviation range reported in previous FE studies and *in vitro* experiments, confirming the validity of the FE model for further analysis.

### Postoperative ROM

**FIGURE 5:**
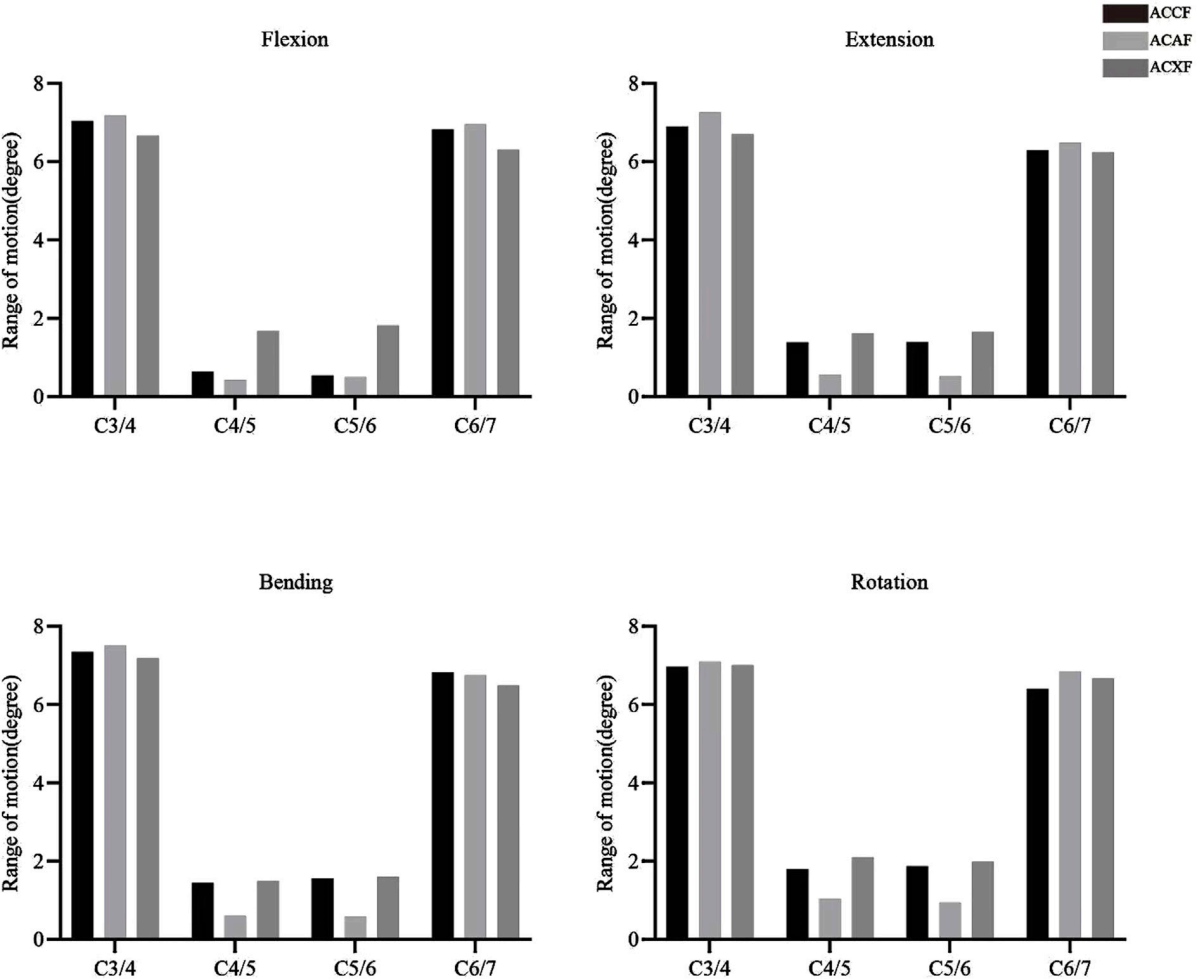
Comparison of postoperative ROMs at C3-7 among ACCF, ACAF, and ACXF.

The postoperative ROMs at C4-C6 for ACCF, ACAF, and ACXF were 1.18°, 0.93°, and 3.50° in flexion; 2.79°, 1.09°, and 3.28° in extension; 3.01°, 1.20°, and 3.10° in lateral bending; and 3.68°, 1.99°, and 4.09° in axial rotation, respectively (Figure 5). Among the surgical methods, ACXF exhibited the highest ROMs, while ACAF showed the lowest. Motion within the fusion region was significantly restricted. Although adjacent segments exhibited compensatory movements, total ROMs remained lower than preoperative levels.

### Internal fixation system stress

**FIGURE 6:**
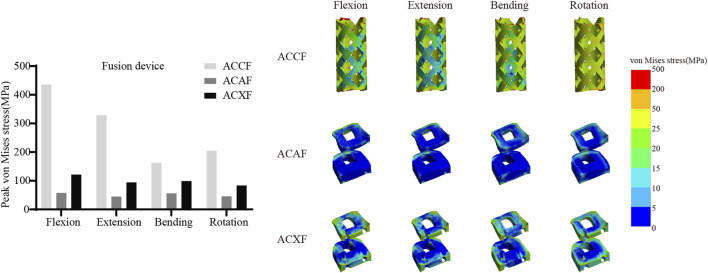
Peak von Mises stresses and distribution cloud maps of fusion devices.

**FIGURE 7:**
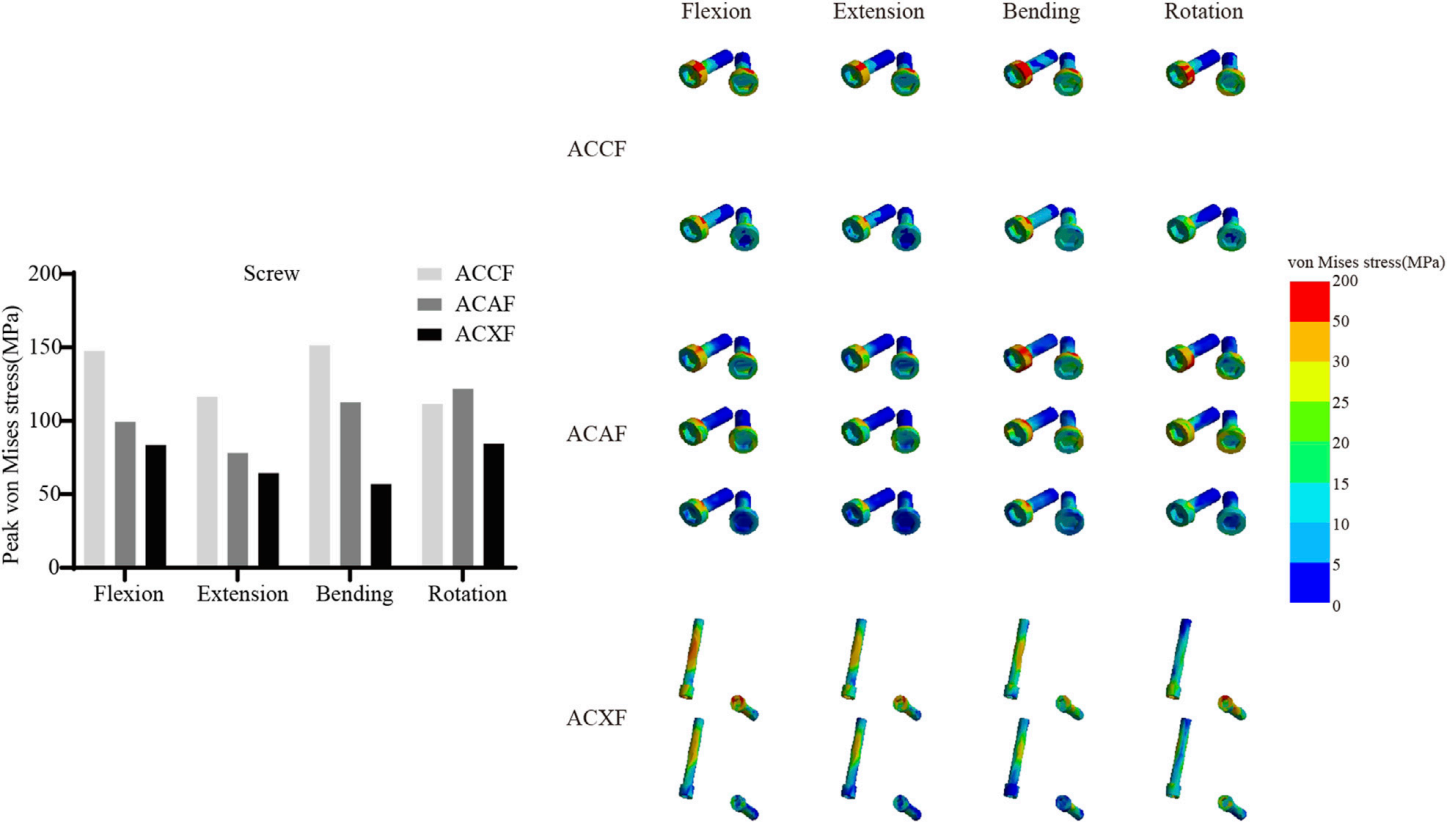
Peak von Mises stresses and distribution cloud maps of screws.

**FIGURE 8:**
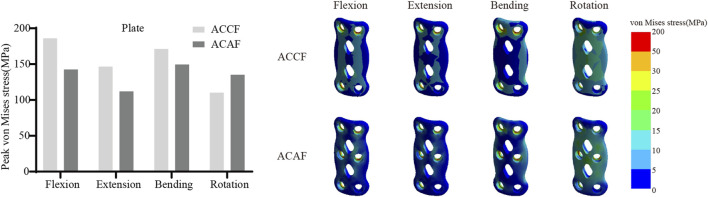
Peak von Mises stresses and distribution cloud maps of plates.

Figure 6 shows the von Mises stresses in the fusion devices. The peak von Mises stresses for ACCF, ACAF, and ACXF were 435.81 MPa, 57.52 MPa, and 121.42 MPa in flexion; 328.64 MPa, 44.92 MPa, and 94.53 MPa in extension; 162.70 MPa, 55.90 MPa, and 99.15 MPa in lateral bending; and 204.75 MPa, 45.74 MPa, and 83.65 MPa in axial rotation, respectively. Figure 7 shows the von Mises stresses in the screws. The peak von Mises stresses for ACCF, ACAF, and ACXF were 147.64 MPa, 99.29 MPa, and 83.60 MPa in flexion; 116.47 MPa, 78.15 MPa, and 64.46 MPa in extension; 151.40 MPa, 112.50 MPa, and 56.85 MPa in lateral bending; and 111.44 MPa, 121.76 MPa, and 84.40 MPa in axial rotation, respectively. Figure 8 shows the von Mises stresses in the plates. The peak von Mises stresses for ACCF and ACAF were 186.08 MPa and 142.52 MPa in flexion; 146.64 MPa and 112.06 MPa in extension; 171.23 MPa and 149.60 MPa in lateral bending; and 110.24 MPa and 135.29 MPa in axial rotation, respectively. The peak stresses of the plant system within ACCF are the highest, especially in the fusion device. ACAF has the lowest cage stresses, while screws have the lowest stresses in ACXF.

### Cortical endplate stress

**FIGURE 9:**
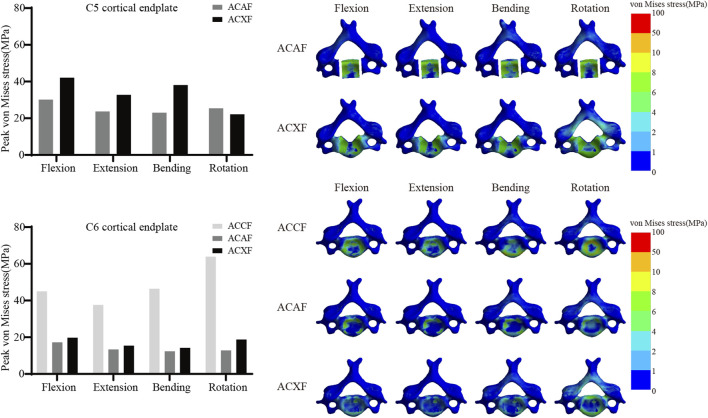
Peak von Mises stresses and distribution cloud maps of C5 and C6 cortical endplates.

Figure 9 shows the von Mises stresses of C5 and C6 cortical endplates. The peak von Mises stresses on the C5 cortical endplate for ACAF and ACXF were 30.19 MPa and 42.11 MPa in flexion, 23.73 MPa and 32.77 MPa in extension, 23.06 MPa and 38.13 MPa in lateral bending, and 25.44 MPa and 22.16 MPa in axial rotation, respectively. The peak von Mises stresses on the C6 cortical endplate for ACCF, ACAF, and ACXF were 45.03 MPa, 17.20 MPa, and 19.74 MPa in flexion; 37.63 MPa, 13.36 MPa, and 15.45 MPa in extension; 46.43 MPa, 12.35 MPa, and 14.22 MPa in lateral bending; and 63.87 MPa, 12.86 MPa, and 18.76 MPa in axial rotation, respectively.

### Intervertebral disc pressure

**FIGURE 10:**
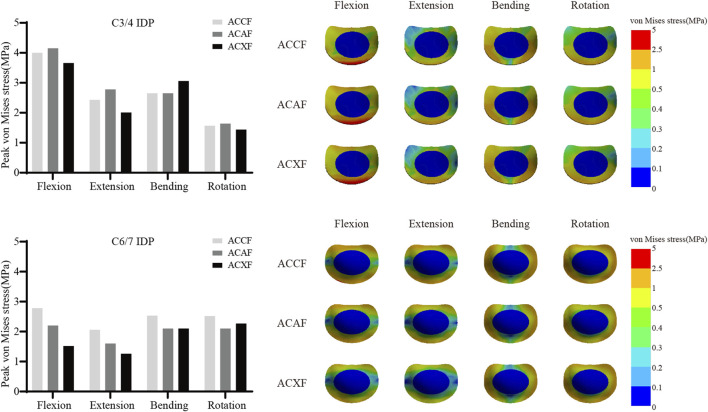
Peak IDPs and distribution cloud maps at C3/4 and C6/7.

Figure 10 shows the IDPs at C3/4 and C6/7. The peak IDPs at C3/4 for ACCF, ACAF, and ACXF were 4.00 MPa, 4.15 MPa, and 3.66 MPa in flexion; 2.43 MPa, 2.78 MPa, and 2.01 MPa in extension; 2.67 MPa, 2.65 MPa, and 3.06 MPa in lateral bending; and 1.57 MPa, 1.64 MPa, and 1.44 MPa in axial rotation, respectively. The peak IDPs at C6/7 for ACCF, ACAF, and ACXF were 2.78 MPa, 2.20 MPa, and 1.52 MPa in flexion; 2.06 MPa, 1.60 MPa, and 1.26 MPa in extension; 2.53 MPa, 2.10 MPa, and 2.08 MPa in lateral bending; and 2.52 MPa, 2.10 MPa, and 2.27 MPa in axial rotation, respectively.

## Discussion

FE analysis, through three-dimensional reconstruction and material modeling, enables the simulation of skeletal stress distribution, joint biomechanics, and prosthesis–bone interface interactions, providing a foundation for personalized surgical planning. Traditionally, for COPLL involving the entire posterior vertebral body, ACCF or posterior approaches were the only viable options. ACXF allows direct exposure and resection of the posterior ossified mass, while ACAF indirectly decompresses the spinal cord by lifting the vertebrae-OPLL complex. Given that both ACAF and ACXF achieve a decompression range comparable to ACCF, they share similar surgical indications, thereby expanding clinical treatment options. This study aims to establish FE models of ACCF, ACAF, and ACXF to investigate potential biomechanical differences, providing insights to inform surgical decision-making.

### Construct stability

ROM is commonly used in FE analysis to objectively assess model stability, with smaller deformations under loading conditions indicating superior performance. As expected, all three surgical models exhibited significantly restricted ROMs compared to the preoperative state, while adjacent segments demonstrated compensatory motion, consistent with previous findings in spinal fusion research (Lin et al., 2024; Shen et al., 2022; Zhang et al., 2022; Wang Y. et al., 2023; Wo et al., 2021). Compared to ACCF, our study found that ACAF led to a more pronounced reduction in ROMs across all directions, aligning with Kong’s FE studies (Kong et al., 2024). This is likely due to ACAF preserving more of the vertebral structure, thereby enhancing the intrinsic mechanical stability of the cervical spine. Both ACAF and ACXF avoid subtotal corpectomy, maintaining spinal structural integrity. Structurally, ACAF utilizes a two-level cage-plate system, whereas ACXF employs a two-level zero-profile system. Multiple studies have reported greater ROM in the zero-profile system compared to the cage-plate system (Panchal et al., 2019; Salari et al., 2022; Zhang X. et al., 2023). Consistently, our results indicate that ACXF exhibits greater ROMs than ACAF, possibly due to the anterior plate in ACXF. Given its ability to maximize vertebral preservation while providing strong internal fixation, ACAF may be a preferable option for patients with compromised baseline cervical stability, such as those with severe osteoporosis, weakened paraspinal musculature, or altered cervical alignment.

### Risks of instrument-related complications

Implant-related complications, including fracture, loosening, and displacement, may result in neck pain, dysphagia, or spinal cord compression, potentially necessitating revision surgery. The peak von Mises stress within the internal fixation system serves as an indicator of mechanical failure risk. It is noteworthy that the screw stresses in ACAF are higher than those in ACXF, whereas the opposite trend is observed for the fusion device. This discrepancy can be attributed to the distinct fixation structures of the two techniques. In ACAF, the plate-screw construct functions as a load-sharing bridge, redistributing axial forces and thereby increasing screw stresses while reducing the stress borne by the cage. In contrast, ACXF relies on zero-profile cages without supplement; the screws in this construct primarily serve as anchors to secure the cage rather than as load-bearing elements. Consequently, the cage in ACXF directly assumes the majority of the axial load transmission, which explains the relatively lower screw stresses but higher stresses within the fusion device. Our study found that ACCF exhibited the highest peak stresses within the fixation system, likely due to the extensive vertebral resection and increased load-bearing demands placed on the implants for structural reconstruction. Clinical follow-up studies have reported implant-related complications following ACCF, whereas no such cases have been documented for ACAF or ACXF to date (Wang Y. et al., 2023; Kong et al., 2024). However, this may be attributed to the limited number of cases and short follow-up duration. Further clinical studies are needed to validate these findings.

### Bone fusion

The internal fixation system plays a crucial role in maintaining early postoperative stability. Effective bony fusion is essential for successful surgery, and fusion rate is a key metric for clinical evaluation. According to Wolff’s law (Frost, 2004), bone formation is optimal when mechanical stress is maintained within the range of 2–60 MPa; stress levels that are too low may lead to bone resorption, while excessive stress can result in bone damage. Stress distribution maps indicate favorable loading patterns across the cortical endplates in ACCF, ACAF, and ACXF, consistent with clinical studies reporting no significant differences in fusion rates among the three techniques within 1 year (Sun et al., 2017; Wang Y. et al., 2023). A region of low stress is observed at the center of the cortical endplate, emphasizing the importance of adequate bone grafting within the fusion construct to ensure sufficient mechanical stimulus for bone growth.

### Subsidence resistance

Subsidence is a common complication following spinal fusion, often attributed to factors such as osteoporosis, endplate damage, cancellous bone exposure, and microfractures. Our analysis revealed that ACCF exhibited the highest cortical endplate stresses, consistent with clinical findings that titanium mesh cages have a higher subsidence rate than interbody cages (Chen et al., 2023; Lee et al., 2021), which is related to the greater load-bearing demands and smaller contact surface. Zhang proposed a novel anatomical titanium mesh cage with integrated spacers (Zhang et al., 2022), which theoretically offers superior biomechanical performance and may represent a promising clinical alternative. Xu compared subsidence across seven cervical fusion systems (Xu et al., 2020), including standalone cage constructs, cage-plate constructs, and zero-profile systems. Their findings demonstrated that the addition of an anterior plate significantly improves resistance to subsidence, which may explain the lower cortical endplate stresses observed in ACAF compared to ACXF. Additionally, within the same surgical approach, the C5 cortical endplate exhibited greater stresses than C6, likely due to osteotomy-induced disruption of vertebral integrity. The preservation of bony structures plays a crucial role in maintaining resistance to deformation.

### Adjacent segment degeneration

Postoperative increases in adjacent segment ROM can lead to further compression or stretching of the intervertebral disc, elevating IDPs and accelerating adjacent segment disease. Among the three surgical models, ACXF demonstrated the lowest adjacent segment IDPs. Notably, ACXF also imposed the least restriction on fusion segment ROMs, which may indirectly reduce compensatory motion at adjacent levels. This finding aligns with previous meta-analyses (Guo et al., 2021; Kahaer et al., 2022; Liu et al., 2022; Chen et al., 2024), which reported a lower risk of adjacent segment disease in zero-profile systems compared to cage-plate constructs. Ouyang did not observe significant differences in the adjacent segment IDPs between the single-level mesh-plate system and the two-level cage-plate system (Ouyang et al., 2020). This further verifies the results and emphasizes the advantages of ACXF in preventing adjacent segment disease. Consistent with prior FE studies (Zhang X. et al., 2023; Zhang et al., 2022), our results show that IDPs are primarily concentrated in the annulus fibrosus, with a distribution pattern corresponding to the direction of movement. The cellular and biomechanical responses of the annulus fibrosus under high stress may provide further insights into intervertebral disc degeneration.

### Limitation

Currently, FE analysis is widely used in biomechanics research, offering valuable insights for both basic medical science and clinical applications. However, this study has several limitations. First, the cervical spine model was based on CT data from a healthy volunteer and may not fully reflect degenerative changes in OPLL patients, including osteoporosis, osteophyte formation, small joint disorders, intervertebral disc aging, and ligament degeneration, etc. Second, most ligaments were modeled as spring elements, which cannot accurately reflect the stiffness and restraint effects of ossified lesions. Third, the simplified cervical spine and implant system may not fully replicate the *in vivo* biomechanical environment. Fourth, most contact interactions in the FE model were defined as tied connections, potentially overlooking certain micromovements. Finally, we acknowledge that ACAF is often clinically indicated for multilevel OPLL, whereas this study simulated only a single-segment model. The biomechanical behavior of multilevel constructs may differ from that of a single-level procedure. A previous FE study comparing single- and two-level anterior cervical discectomy and fusion found that, after adding additional segments, the biomechanical differences between zero-profile and cage-plate systems not only persisted but were further amplified (Hua et al., 2020). Multilevel studies are therefore warranted to achieve a more comprehensive understanding of biomechanical performance in clinical practice. Thus, this study aims to identify biomechanical trends rather than establish definitive conclusions.

## Conclusion

The risks associated with internal fixation in ACAF and ACXF are relatively low, with both approaches demonstrating strong resistance to subsidence. ACAF provides superior overall stability and may therefore be more suitable for patients with poor cervical stability, such as those with severe degenerative changes or osteoporosis. In contrast, ACXF is associated with a lower risk of adjacent segment disease and may be preferable for younger patients who require preservation of cervical mobility over the long term. Taken together, these findings suggest that both ACAF and ACXF can serve as preferable alternatives to ACCF, and surgical strategies should be tailored to individual patient characteristics.

## Data Availability

The original contributions presented in the study are included in the article/supplementary material, further inquiries can be directed to the corresponding author.
